# Exploring the prognostic value of EBV DNA in advanced nasopharyngeal carcinoma treated with chemoradiotherapy using AI-based modeling

**DOI:** 10.3389/fonc.2025.1650377

**Published:** 2025-09-12

**Authors:** Yang Yang, Ningchuan Shang, Shun Lu, Lintao Li, Peng Xu, Xianliang Wang, Fan Li, Yue Su, Yuan Qin, Jinyi Lang, Jie Zhou

**Affiliations:** ^1^ Department of Oncology, The Third People’s Hospital of Chengdu, Chengdu, China; ^2^ School of Clinical Medicine, Sichuan College of Traditional Chinese Medicine, Mianyang, China; ^3^ Department of Radiation Oncology, Precision Radiation in Oncology Key Laboratory of Sichuan Province, Sichuan Clinical Research Center for Cancer, Sichuan Cancer Hospital & Institute, Sichuan Cancer Center, School of Medicine, University of Electronic Science and Technology of China, Chengdu, China; ^4^ Department of Otorhinolaryngology Head and Neck Surgery, Chongqing General Hospital, Chongqing University, Chongqing, China

**Keywords:** EBV DNA, advanced nasopharyngeal carcinoma, prognostic value, artificial intelligence, machine learning, chemoradiotherapy

## Abstract

**Background:**

Epstein–Barr virus (EBV) DNA is a well-established biomarker in nasopharyngeal carcinoma (NPC), but its integration into artificial intelligence (AI)–based prognostic tools remains limited. This study aimed to develop and validate AI models incorporating EBV DNA load levels to predict progression-free survival (PFS) in patients with advanced NPC treated with concurrent chemoradiotherapy (CRT).

**Methods:**

A retrospective multicenter cohort of 503 patients was divided into training (n = 301) and validation (n = 202) sets. Four machine learning algorithms—Cox regression, LASSO, RSF, and GBM—were applied to predict 1- and 1.5-year PFS in patients with advanced NPC. Model performance was evaluated using the concordance index (C-index), time-dependent receiver operating characteristic (ROC), decision curve analysis (DCA), and interpretability tools such as SHAP values and partial dependence plots (PDP).

**Results:**

The 1-, 3-, and 5-year PFS rates were 100.0%, 91.5%, and 88.6% in the EBV = 0 group; 99.4%, 91.2%, and 88.5% in the > 0 and < 1500 group; and 92.3%, 81.0%, and 75.7% in the ≥ 1500 group, respectively, with statistically significant differences among the three groups (*P* = 0.0024). The RSF model outperformed other models with the highest C-index (0.778) and area under the ROC curve of 0.810 and 0.634 at 1 and 1.5 years, respectively. EBV DNA emerged as the most influential predictor across all interpretability analyses. Patients with EBV DNA ≥1500 copies/ml had the poorest predicted survival, showing a distinct threshold effect in the PDP.

**Conclusions:**

High EBV DNA levels were associated with poorer PFS in advanced NPC. Among the models evaluated, the RSF model demonstrated the best predictive performance and interpretability. EBV-informed AI modeling represents a promising approach for enhancing individualized risk prediction and clinical decision-making in NPC.

## Introduction

Nasopharyngeal carcinoma (NPC) is a malignancy of the head and neck region with high prevalence in east and Southeast Asia, and its development is strongly linked to Epstein–Barr virus (EBV) infection (1, 2). Although intensity-modulated radiotherapy (IMRT) combined with chemotherapy has become the standard treatment for advanced-stage disease, recurrence and metastasis remain major clinical challenges (3). Plasma EBV DNA has emerged as a valuable biomarker for estimating tumor burden and predicting prognosis, however, its accuracy and consistency in different clinical scenarios are still debated (4, 5).

Conventional regression-based approaches may not fully uncover the intricate relationships between EBV levels and various prognostic factors. In contrast, artificial intelligence (AI) techniques, especially machine learning models, provide a powerful framework for handling complex datasets and capturing nonlinear associations (6–8). These models have shown increasing promise in cancer prognosis and treatment optimization (9, 10). Nonetheless, few studies have systematically evaluated the integration of EBV DNA into AI-based prediction tools for NPC.

In this study, we propose to develop a robust and interpretable AI model incorporating EBV DNA levels and clinical characteristics to enhance survival prediction in patients undergoing concurrent chemoradiotherapy. This model aims to assist clinicians in tailoring personalized treatment strategies and long-term monitoring plans.

## Materials and methods

### Study design and participants

From March 2012 to May 2020, 503 patients with advanced-stage NPC who received concurrent chemoradiotherapy (CRT) were retrospectively reviewed from four hospitals in China. Inclusion criteria for this study were as follows: (1) histologically confirmed nasopharyngeal carcinoma; (2) stage III–IV disease; (3) received first-line concurrent CRT as the initial treatment; (4) complete baseline EBV DNA measurements before treatment; (5) complete follow-up and survival data available. Exclusion criteria included: (1) history of prior malignancy or previous anti-cancer treatment; (2) with serious complications or any other serious chronic diseases; (3) unable to complete the treatment. This study was approved by the institutional review board and ethics committees (KY S2023-081-01). As this was a retrospective study, the informed consent was waived by the Ethics Committee. Participant information is confidential, and the study was conducted in accordance with the Declaration of Helsinki.

### Treatments

All enrolled patients underwent IMRT alongside synchronous chemotherapy based on platinum compounds. The delineation of gross tumor volume (GTV) included two components: the nasopharyngeal lesion itself (GTVnx) and lymph nodes confirmed as metastatic through clinical or radiologic evaluation (GTVnd), primarily based on MRI and/or PET-CT findings. To capture areas potentially harboring microscopic disease, clinical target volumes (CTV) were contoured by expanding the GTVs to incorporate adjacent high-risk tissues. Planning target volumes (PTV) were created by applying an additional 3 – 5 mm margin around each CTV, accounting for patient movement and setup variation during treatment sessions. A total radiation dose of 70 Gy was prescribed, aimed at ensuring that no less than 95% of the PTV received the full dose. The course was delivered over 30 to 33 fractions, typically completed within a period of 6 to 7 weeks.

### Endpoints and follow-up

Progression-free survival (PFS) was defined as the interval from the initiation of treatment to either documented disease progression, as assessed by imaging according to RECIST criteria, or death from any cause, whichever occurred first. Disease progression was evaluated based on institutional radiology reports without central imaging review, reflecting real-world clinical practice across participating centers.

### AI-model

A retrospective dataset comprising 503 patients was split into two groups using a 6:4 random allocation strategy, resulting in a training cohort of 301 cases and a validation cohort of 202 cases. The training set was used to construct predictive models for progression-free survival (PFS) at both 1 and 1.5 years. The following six baseline variables were incorporated as potential prognostic indicators: sex, age, T stage, N stage, clinical stage, and EBV DNA levels at diagnosis.

Four modeling algorithms were employed: Cox proportional hazards model, least absolute shrinkage and selection operator (LASSO) regression, random survival forest (RSF), and gradient boosting machine (GBM). Each model’s discriminative performance was assessed via concordance index (C-index) metrics.

Model performance was subsequently evaluated in the independent validation cohort using time-dependent receiver operating characteristic (ROC) curves and decision curve analysis (DCA). To further elucidate the underlying mechanics of the models, explainability methods such as SHAP values, partial dependence survival plots (PDP), time-dependent feature importance analysis, and Brier scores were applied in the training group.

### Statistical analyses

Categorical variables were compared using the chi-square test. The Kaplan–Meier method was used to estimate PFS, and survival differences between groups were assessed by the log-rank test. All statistical analyses related to AI-based modeling were performed using R software.

## Results

### Baseline characteristics

The cohort was divided into three groups: EBV DNA = 0 copies/ml (n = 120), > 0 and < 1500 copies/ml (n = 173), and ≥ 1500 copies/ml (n = 210). There were no statistically significant differences among the groups in terms of age (p = 0.762) or sex (*p* = 0.967). However, significant differences were observed in T stage distribution (*p* = 0.004). The proportion of T4 disease increased with rising EBV DNA levels, from 10.0% in the 0 group to 24.3% in the ≥ 1500 group. Similarly, N stage was significantly associated with EBV DNA levels (*p* < 0.001). The frequency of N3 involvement was markedly higher in the ≥ 1500 group (21.4%) compared to the 0 group (7.5%). A significant shift in clinical stage was also observed (*p* < 0.001), with stage IV disease more prevalent among patients with high EBV DNA (41.0%) compared to those with undetectable levels (16.7%, **Table 1**).

**Table 1:** Baseline clinical characteristics stratified by EBV DNA levels.

Characteristic	All	Pre-EBV DNA			P
		0	> 0, < 1500	≥ 1500	
	503	120	173	210	
Age					0.762
< 45	218 (43.3%)	54 (45.0%)	77 (44.5%)	87 (41.4%)	
≥ 45	285 (56.7%)	66 (55.0%)	96 (55.5%)	123 (58.6%)	
Sex					0.967
Female	139 (27.6%)	33 (27.5%)	49 (28.3%)	57 (27.1%)	
Male	364 (72.4%)	87 (72.5%)	124 (71.7%)	153 (72.9%)	
T Stage					0.004
T1	7 (1.39%)	2 (1.67%)	2 (1.16%)	3 (1.43%)	
T2	45 (8.95%)	13 (10.8%)	15 (8.67%)	17 (8.10%)	
T3	371 (73.8%)	93 (77.5%)	139 (80.3%)	139 (66.2%)	
T4	80 (15.9%)	12 (10.0%)	17 (9.83%)	51 (24.3%)	
N Stage					< 0.001
N0	45 (8.95%)	22 (18.3%)	17 (9.83%)	6 (2.86%)	
N1	171 (34.0%)	46 (38.3%)	56 (32.4%)	69 (32.9%)	
N2	215 (42.7%)	43 (35.8%)	82 (47.4%)	90 (42.9%)	
N3	72 (14.3%)	9 (7.50%)	18 (10.4%)	45 (21.4%)	
Stage					< 0.001
III	362 (72.0%)	100 (83.3%)	138 (79.8%)	124 (59.0%)	
IV	141 (28.0%)	20 (16.7%)	35 (20.2%)	86 (41.0%)	

### Survival outcomes

**Figure 1:**
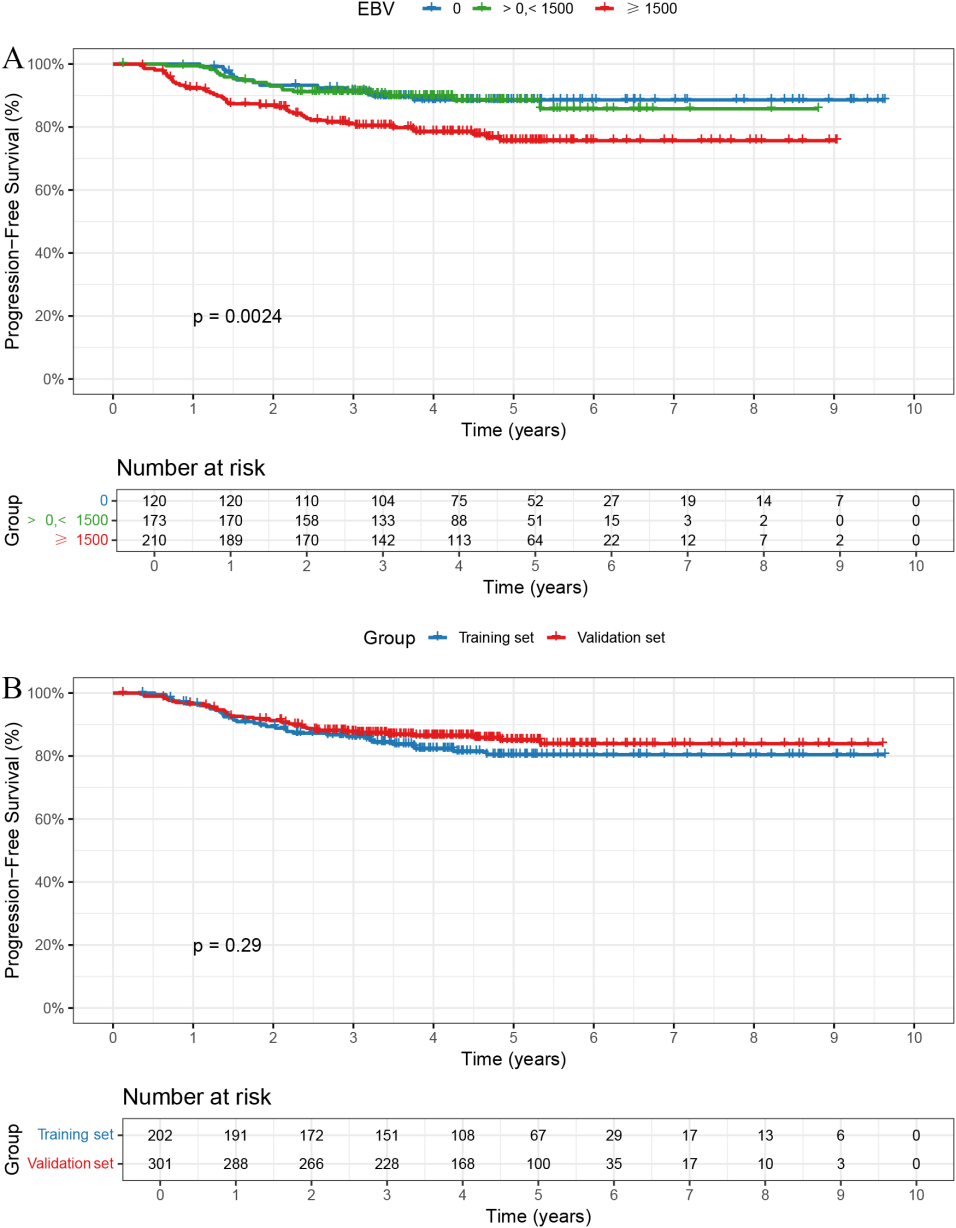
Kaplan-Meier curves for PFS. **(A)** Patients with EBV DNA ≥1500 copies/ml had significantly worse PFS (*p* = 0.0024). **(B)** No significant PFS difference between training and validation sets (*p* = 0.29).

The follow-up duration for the cohort was consistently recorded. The median follow-up time was 4.59 years, with follow-up data collected through telephone follow-ups and hospital records. Patients were censored at the time of the last follow-up or death, whichever occurred first. In the overall cohort, the 1-, 3-, and 5-year PFS rates were 96.60%, 87.10%, and 83.19%, respectively. The 1-, 3-, and 5-year PFS rates were 100.0%, 91.5%, and 88.6% in the EBV = 0 group; 99.4%, 91.2%, and 88.5% in the > 0 and < 1500 group; and 92.3%, 81.0%, and 75.7% in the ≥ 1500 group, respectively, with statistically significant differences among the three groups (**Figure 1A**, *P* = 0.0024).

### AI-model construction

Baseline characteristics were well balanced between the training and validation sets, except for sex, which showed a statistically significant difference (**Table 2**). The 1-, 3-, and 5-year PFS rates were 96.66%, 87.72%, and 85.03% in the training set, and 96.50%, 86.15%, and 80.43% in the validation set, respectively, with no significant differences observed between the two groups (**Figure 1B**, *P* = 0.29).

**Table 2:** Comparison of baseline clinical characteristics between training and validation sets.

Characteristic	Validation set	Training set	P
	202	301	
Age			0.363
< 45	93 (46.0%)	125 (41.5%)	
≥ 45	109 (54.0%)	176 (58.5%)	
Sex			0.003
Female	71 (35.1%)	68 (22.6%)	
Male	131 (64.9%)	233 (77.4%)	
T Stage			0.177
T1	0 (0.00%)	7 (2.33%)	
T2	18 (8.91%)	27 (8.97%)	
T3	151 (74.8%)	220 (73.1%)	
T4	33 (16.3%)	47 (15.6%)	
N Stage			0.965
N0	17 (8.42%)	28 (9.30%)	
N1	71 (35.1%)	100 (33.2%)	
N2	85 (42.1%)	130 (43.2%)	
N3	29 (14.4%)	43 (14.3%)	
Stage			0.56
III	142 (70.3%)	220 (73.1%)	
IV	60 (29.7%)	81 (26.9%)	
EBV DNA			0.757
0	48 (23.8%)	72 (23.9%)	
> 0, < 1500	66 (32.7%)	107 (35.5%)	
≥ 1500	88 (43.6%)	122 (40.5%)	

In the training cohort, six clinical variables—sex, age, T stage, N stage, clinical stage, and EBV DNA levels—were selected to construct four prognostic models using different machine learning approaches. The C-index were 0.740 for the Cox model, 0.707 for the LASSO model, 0.778 for the RSF, and 0.732 for the GBM.

### AI model validation

**Figure 2:**
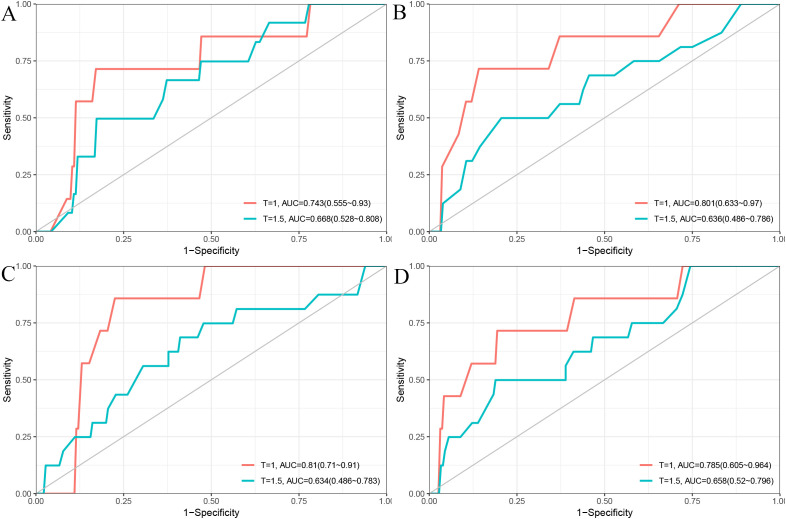
Time-dependent ROC curves for 1-year and 1.5-year PFS prediction using different models. **(A)** Cox model; **(B)** LASSO model; **(C)** RSF model; **(D)** GBM model.

**Figure 3:**
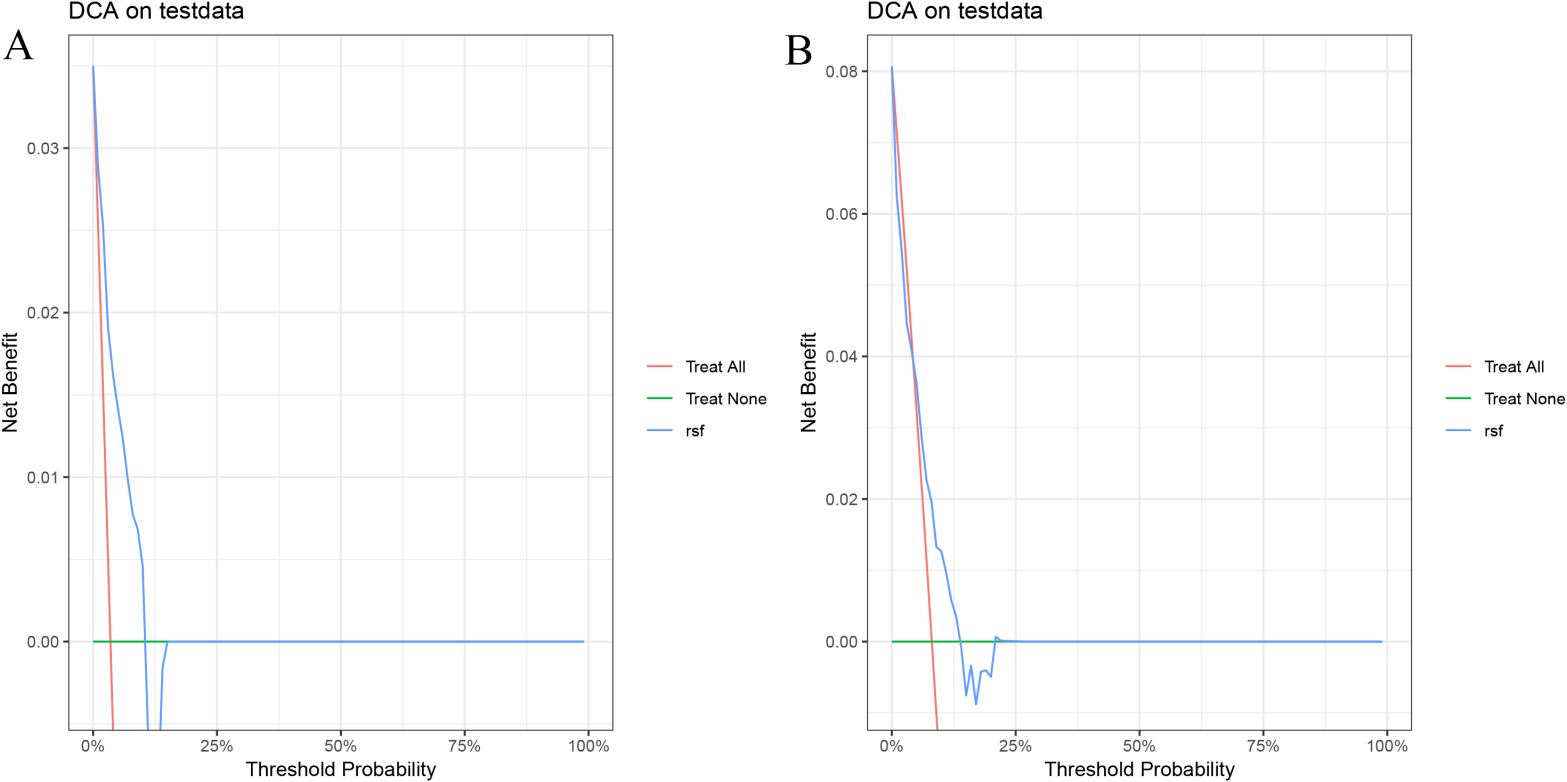
Decision curve analysis of the RSF model on the test set. **(A)** 1-year PFS; **(B)** 1.5-year PFS.

In the validation cohort, the 1- and 1.5-year area under the ROC curve (AUC) were 0.743 and 0.668 for the Cox model (**Figure 2A**), 0.801 and 0.636 for the LASSO model (**Figure 2B**), 0.810 and 0.634 for the RSF model (**Figure 2C**), and 0.785 and 0.658 for the GBM model (**Figure 2D**). The DCA demonstrated that the RSF model provided favorable net clinical benefit and showed good stability across different threshold probabilities (**Figures 3A, B**).

**Figure 4:**
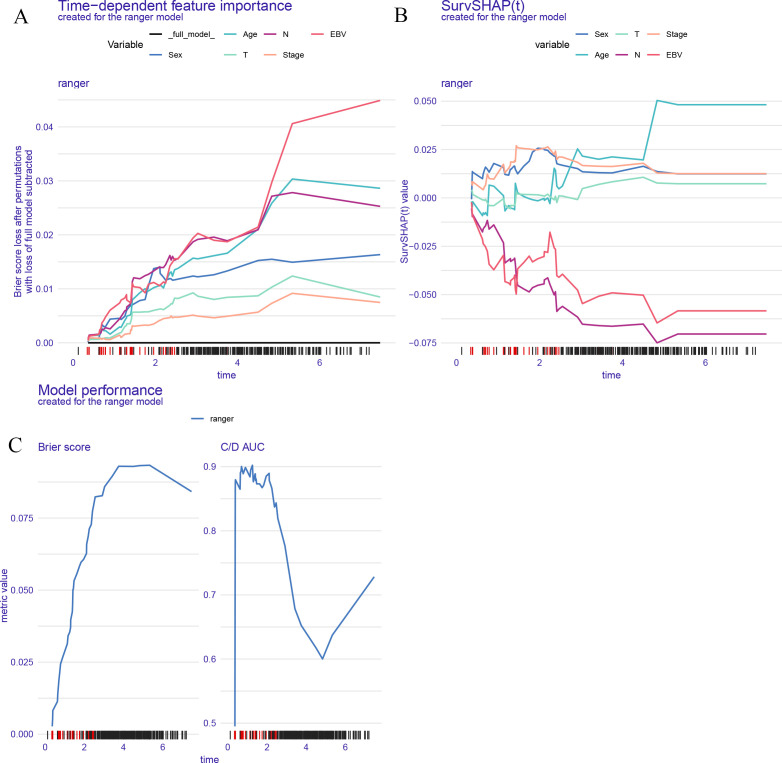
Model interpretability and performance of the RSF model. **(A)** Time dependent feature importance shows EBV DNA and N stage had the greatest impact on model performance over time. **(B)** SurvSHAP values illustrate the dynamic contribution of each variable to individual risk prediction. **(C)** Brier score and time dependent C/D AUC demonstrate the model's predictive accuracy and discrimination ability over time.

**Figure 5:**
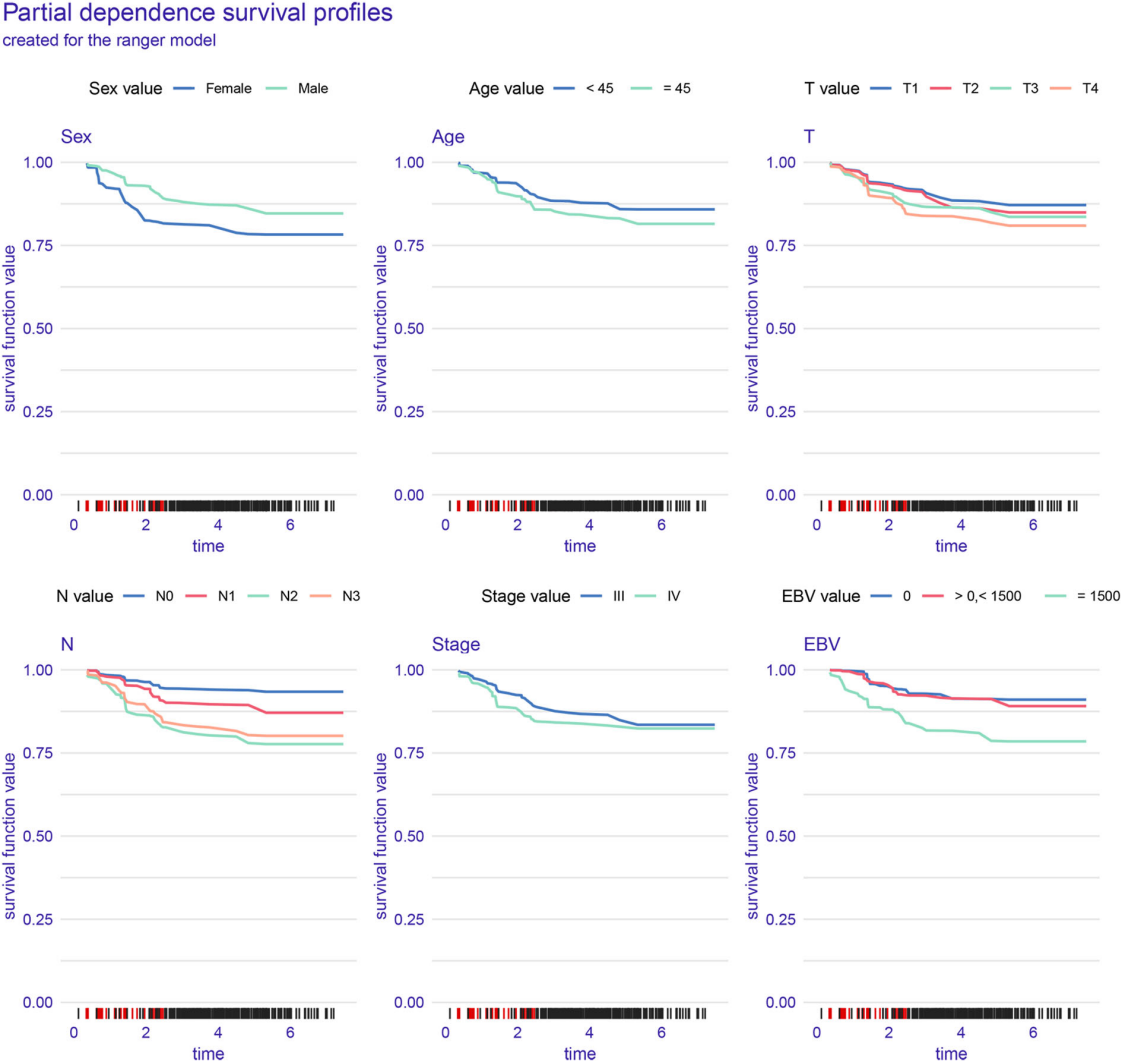
Partial dependence survival curves of key variables in the RSF model.

**Figure 4** illustrates the interpretability and performance metrics of the random survival forest (RSF) model in the training cohort. The time-dependent feature importance analysis (**Figure 4A**) showed that EBV DNA level and N stage were the most influential predictors throughout the entire follow-up period. The SHAP plot further demonstrated that both EBV and N stage were positively associated with increased risk (**Figure 4B**). Model performance was stable over time, as reflected by a consistently low Brier score and a time-dependent concordance index (C/D AUC) that remained above 0.8 for a substantial portion of the follow-up (**Figure 4C**). The partial dependence plot (PDP) showed that patients with EBV DNA ≥1500 copies/ml had the lowest predicted survival throughout the follow-up period (**Figure 5**).

## Discussion

In recent years, EBV DNA has emerged as a key biomarker in the management of NPC, particularly in regions with a high disease burden such as Southeast Asia (11–13). While its prognostic relevance is well established, translating EBV DNA levels into individualized survival predictions remains a challenge. In this study, we sought to address this gap by constructing AI–based models that integrate EBV DNA with standard clinical features to predict PFS in advanced NPC patients treated with concurrent CRT.

Unlike traditional statistical methods, which typically assume linear associations and fixed hazard ratios, AI algorithms are capable of modeling more complex, nonlinear interactions (14, 15). This advantage is particularly relevant in heterogeneous cancers such as NPC, which clinical behavior is influenced by both tumor burden and host factors (16). By incorporating machine learning techniques—namely Cox regression, LASSO, RSF, and GBM—we aimed to compare the predictive capacity of conventional and data-driven approaches (17–20).

Among the four models tested, the RSF algorithm demonstrated the strongest performance (21). It achieved the highest C-index in the training cohort and showed stable results in the validation set. Beyond overall accuracy, interpretability analyses—including SHAP values, time-dependent feature importance, and partial dependence plots—consistently highlighted EBV DNA as the most significant predictor of survival outcomes. These findings reinforce the notion that EBV DNA is not merely a passive biomarker, but an active contributor to risk stratification in AI-driven models.

The partial dependence plot for EBV DNA revealed a clear inflection point: patients with baseline EBV DNA ≥1500 copies/ml consistently showed markedly lower predicted survival probabilities. Interestingly, survival curves for patients with undetectable or low-level EBV DNA copies were similar to those of patients with EBV DNA > 0 and < 1500 copies/ml, suggesting a threshold effect. These observations are in line with previous clinical studies and further validate the value of integration of EBV DNA into automated risk prediction frameworks.

Another strength of this study lies in its focus on model interpretability. One of the major criticisms of AI in healthcare is the “black box” nature of many algorithms (22, 23). By applying tools such as SHAP and PDP, we made our models more transparent and clinically meaningful (24). These visualizations allow clinicians understanding not only that EBV matters, but how and when it exerts the greatest impact on patient outcomes.

In our study, the RSF model demonstrated superior performance compared to other machine learning algorithms. RSF is an ensemble learning method that is particularly well-suited for survival analysis due to its ability to handle both censored data and complex non-linear relationships between predictors and survival outcomes (25, 26). Unlike traditional survival models, RSF does not require the assumption of proportional hazards and can model interactions between variables more effectively. Additionally, RSF can handle high-dimensional datasets with many variables and is less prone to overfitting compared to other algorithms, making it a robust choice for survival prediction in clinical datasets like ours (27).

From a clinical standpoint, integrating EBV DNA into AI-based models offers more than statistical insight—it provides a practical tool for precision medicine (28–30). By accurately identifying patients at higher risk of disease progression, clinicians can consider early treatment intensification, closer surveillance, or inclusion in clinical trials. Conversely, patients with low-risk profiles may benefit from treatment de-escalation, reducing toxicity and preserving quality of life. The model’s transparency also enhances clinical confidence, making it more acceptable for integration into multidisciplinary tumor boards. As such, EBV-informed AI models may serve as decision-support systems that personalize management strategies and improve long-term outcomes in advanced NPC (31–33).

Although the current model demonstrates promising results, integrating other modalities such as radiomics, genomic alterations, and immune-related biomarkers could further improve its prognostic performance. Radiomics could provide detailed imaging features related to tumor phenotype, allowing for more accurate risk stratification. Genomic alterations, including mutations, copy number variations, and methylation patterns, could offer insights into the underlying molecular mechanisms of tumor progression and treatment resistance (34). Additionally, immune-related biomarkers, such as tumor-infiltrating lymphocytes or checkpoint markers, may help capture the immune response to therapy, which is often a critical factor in cancer prognosis (35). Future studies incorporating these multimodal data types, in combination with EBV DNA, would likely improve the model’s accuracy and utility, providing more comprehensive and personalized predictions for patients with advanced NPC.

Despite its strengths, this study has several limitations. First, the retrospective nature of the analysis, even though based on a multicenter cohort, may introduce selection and information bias. Second, while EBV DNA testing was performed uniformly within each center, inter-institutional variability in laboratory procedures cannot be completely ruled out. Third, although treatment protocols were broadly standardized (IMRT with platinum-based concurrent chemotherapy), subtle differences in chemotherapy regimens, supportive care, or radiation planning across centers may contribute to treatment heterogeneity, potentially confounding survival outcomes. Fourth, the model did not incorporate other potentially informative modalities such as radiomics, genomic alterations, or immune-related biomarkers, which could further enhance predictive accuracy. Finally, the absence of external validation limits the generalizability of the model. Future prospective studies should include external datasets and integrate multimodal information to validate and refine AI-based prognostic models for advanced NPC.

## Conclusion

In conclusion, our study demonstrates that elevated EBV DNA levels are closely associated with poorer PFS in patients with advanced NPC. By integrating EBV DNA with advanced AI algorithms, particularly random survival forests, we were able to more effectively characterize its prognostic impact across the disease course. These findings highlight the potential of EBV-informed AI models as valuable tools for enhancing risk stratification and guiding personalized treatment decisions in clinical practice.

## Data Availability

The raw data supporting the conclusions of this article will be made available by the authors, without undue reservation.
